# Occurrence and Residue Concentration of Coccidiostats in Feed and Food of Animal Origin; Human Exposure Assessment

**DOI:** 10.3390/foods8100477

**Published:** 2019-10-11

**Authors:** Rossana Roila, Raffaella Branciari, Ivan Pecorelli, Elisa Cristofani, Cristiano Carloni, David Ranucci, Laura Fioroni

**Affiliations:** 1 Department of Veterinary Medicine, University of Perugia, Via San Costanzo 4, 06126 Perugia, Italy; rossana.roila@studenti.unipg.it (R.R.); david.ranucci@unipg.it (D.R.); 2 Istituto Zooprofilattico Sperimentale dell’Umbria e delle Marche “Togo Rosati”, Via G. Salvemini 1, 06126 Perugia, Italy; i.pecorelli@izsum.it (I.P.); e.cristofani@izsum.it (E.C.); c.carloni@izsum.it (C.C.); l.fioroni@izsum.it (L.F.)

**Keywords:** coccidiostats, carry-over, ADIs, dietary exposure, eggs, meat, LC-MS/MS

## Abstract

Occurring central Italy, 262 unmedicated feed samples and 353 samples of animal tissues and eggs are tested for coccidiostats between 2012 and 2017. A validated multi-residue HPLC-MS/MS method is applied for the simultaneous determination of the 11 coccidiostats licensed in the EU. The dietary exposure to coccidiostats through poultry meat and eggs is calculated for high consumers, and the contribution to acceptable daily intake of coccidiostats is evaluated. The occurrence of positive feed samples ranges from 17.2% in 2012 to 28.3% in 2017, with an average percentage of positive samples of 25%, while 3.8% of feed samples are non-compliant with a concentration ranging from 0.015 mg/kg for diclazuril to 56 mg/kg for narasin. Positive samples of animal tissues, on average, are 34.7%, fully compliant, while 16% of eggs are positive and violative residues are found in 2%. These noncompliant samples show a concentration varying from 2.4 µg/kg to 1002 µg/kg. The contribution of poultry meat and egg consumption to the acceptable daily intake of each coccidiostat is below 1%, highlighting a low direct risk to public health.

## 1. Introduction

Coccidiostats are pharmacologically active molecules employed, since 1940, to prevent and inhibit parasitic protozoa of the genus *Eimeria*, referred to as coccidia, causing a very contagious disease of the gastrointestinal tract in many farmed animals [1,2]. Coccidiosis represents a major disease in poultry, causing intestinal lesions, scarce weight gain, poor feed conversion and poor egg production; in its acute form, coccidiosis causes high mortality rates [3,4,5,6,7]. Aside from intensively farmed animals, such as poultry, coccidiosis also affects extensively reared species including sheep, cattle, pigs and rabbits [2]. Coccidiostats are authorized as feed additives for target animal species by European legislation [8] and can be categorized as polyether ionophores produced by the Streptomycetaceae bacteria family, such as monensin (MON), narasin (NAR), lasalocid (LAS), salinomycin (SAL), semduramicin (SEM) and maduramicin (MAD), or as non-ionophoric synthetic molecules such as halofuginone (HFG), robenidine (ROB), diclazuril (DIC), decoquinate (DEC) and nicarbazin (NIC) [1,2]. Each coccidiostat has individual toxicological characteristics which are based on the molecular mechanisms of action that affect trans-membrane ion transport in the case of the ionophoric compounds. The non-ionophoric compounds represent a very heterogeneous group of molecules which have partially unknown mechanisms of action. However, these mechanisms are not only efficient against coccidians, but also can act against mammalian cells, primarily on cardiac and peripheral muscle tissues. Clinical signs of acute rhabdomyolysis, such as muscle weakness and myocardial insufficiency, have been observed in a small number of cases of acute intoxications with monensin and other ionophoric compounds, both in animals and humans, due to accidental ingestion of the pure compounds [9,10,11,12]. The authorization of coccidiostats as feed additives is based on studies that demonstrate the safety of their use in relation to the target species at the highest proposed levels of incorporation in feed or water, and at multiples of that level to establish a margin of safety [13]. However, during the production of feed containing coccidiostats, unintentional transfer from target to non-target feed (feed for which the use of coccidiostats is not authorized) may occur [14,15]. This unavoidable transfer, referred to as cross-contamination or carry-over, can occur primarily during feed production, but also during transport and storage [16]. Therefore, for the purpose of enabling the feed manufacturer to manage unavoidable carry-over, to ensure animal health, and to contain the risk to consumers, maximum carry-over levels have been established by Commission Regulation European Union (EU) No 574/2011 [17]. The limits have been fixed at 1% of the authorized maximum content in feed for sensitive non-target animal species, in feed used for the period before slaughter and in non-target feed for ‘continuous food-producing animals’ such as dairy cows or laying hens, and 3% in feed for less sensitive non-target animal species. The occurrence of unavoidable carry-over of coccidiostats may result in the presence of residues of these substances in food products of animal origin [2,18]. Therefore, to protect public health, the EU has established maximum levels for the presence of coccidiostats in food of animal origin originating from non-target animal species [19,20,21]. Moreover, EU countries have established surveillance programs to monitor and prevent unacceptable contamination of animal products for human consumption. The surveillance of coccidiostat residues in food has been focused mainly on ionophores and NIC in eggs and poultry meat [2] for a long time, probably related to limitations attributable to analytical methods. The availability of multi-residue analytical methods, suitable for the simultaneous determination of a wider range of coccidiostat residues in a wider range of species tissue, represents an undisputable advantage in the frame of official control [2,22,23,24].

Although some acute toxicity cases in humans, due to the ingestion of pure molecules, have been registered [9,10,11,12], major concerns are addressed toward chronic toxicity due to long-term exposure to low coccidiostat levels [7]. The European Food Safety Authority (EFSA) and the Joint FAO/WHO Expert Committee on Food Additives (JECFA) established, on the basis of toxicological data, acceptable daily intakes (ADIs) for all regulatory anticoccidials, ranging from the lowest values for HFG (0.00003 mg/kg body weight/day) to the highest values for NIC (0.2 mg/kg body weight/day) [25]. A validated and reliable multi-residue method is applied in the present study to investigate the carry-over contamination of regulated ionophoric and non-ionophoric anticoccidials in feed and food of animal origin collected within the context of the Italian official control plans in recent years. A risk assessment concerning dietary exposure to anticoccidials also is performed for all age populations.

## 2. Material and Methods

### 2.1. Sample Collection

The samples were collected by veterinary inspectors in the Umbria and Marche regions (central Italy) within the framework of official control, according to the national residue control plan (NRCP) for feed. Sampling was performed according to the requirements of EU Regulation No 152/2009 [26] amended by Regulation (EU) No 691/2013 [27]. The number of samples was calculated every year, taking into consideration animal production and the number of non-compliant results detected within the preceding year. The samples were sent to the Istituto Zooprofilattico Sperimentale dell’Umbria e delle Marche ‘Togo Rosati’ to perform analyses. Feed samples were finely ground and stored at +4 °C, while egg and muscle samples were homogenized and stored at −20 °C until the day of the analysis.

### 2.2. Standards and Reagents

Standards: LAS A sodium salt (100 μg/mL acetonitrile solution), ROB hydrochloride, SAL monosodium salt hydrate, nigericin sodium salt (ISTD), NIC, NAR, MAD ammonium and MON sodium salt hydrate analytical standards were purchased from Sigma-Aldrich, now Merck KGaA, (Darmstadt, Germany); DIC analytical standard was purchased from SPECTRA 2000 Srl (Rome, Italy); DIC-methyl (ISTD) and DEC analytical standards were purchased from LGC Standards (Milan, Italy); SEM sodium was kindly provided by the Community Reference Laboratory for coccidiostats in Berlin, Germany; and DEC-d5 (ISTD), DNC-d8 (ISTD), HFG hydrobromide, HFG-^13^C_6_ (ISTD) hydrobromide and ROB-d8 hydrochloride analytical standards were purchased from Witega (Berlin, Germany).

Reagents: Acetonitrile, methanol (hypergrade for LC-MS) and ethanol were supplied by Merck KGaA (Darmstadt, Germany); formic acid (99%), dimethyl sulphoxide and ammonia were of analytical grade, and sodium acetate trihydrate was purchased from Carlo Erba (Milan, Italy). Demineralized water was HPLC grade obtained from a Milli-Q purification system (Merck KGaA, Germany). PTFE filters (0.45 μm) were from Merck KGaA (Darmstadt, Germany). SPE silica cartridges (500 mg, 6 mL) were purchased from Biotage (Uppsala, Sweden).

### 2.3. Analytical Methods

#### 2.3.1. Feed and Food Sample Preparation

Feed samples were prepared according to Moretti et al. [23]. Briefly, 5 g of sample was weighed in a 50 mL polypropylene centrifuge tube, spiked with 250 µL of IS solution (1 μg/mL) and allowed to stand for about 15 min. Then, 25 mL of ethanol was added and the mixture was shaken for 30 min on a mechanical agitator. The supernatant was collected in a clean 50 mL polypropylene centrifuge tube. Extraction with ethanol was repeated twice, and the two extracts were combined. The silica cartridge, used as a filter, was conditioned with 5 mL ethanol and loaded with 10 mL of sample extract and 2 mL acetonitrile; the eluate was collected directly in a 15 mL polypropylene centrifuge tube. The eluate was partially dried and then the analytes were quantitatively collected with 6 mL of an ethanol–water–ammonia (90 + 10 + 0.5 *v*/*v*/*v*) solution. The eluates were combined and evaporated to dryness (N_2_, 50 °C). Finally, the dry residues were redissolved in 1 mL methanol, filtered through a 0.45 μm PTFE filter, and transferred into an LC vial.

Regarding food, a similar analytical procedure was applied [22,23]. Two grams of homogenized whole eggs or muscle was weighed in a 50 mL polypropylene centrifuge tube. Fifty microlitres of the same IS solution described for feed was added. The samples were vortex-mixed and allowed to equilibrate for 10 min. Afterwards, 6 mL of acetonitrile and about 4 g of anhydrous sodium sulphate were added; the mixture was vigorously mixed and then centrifuged (4000 rpm, 10 min). The supernatant was collected in a clean 50 mL polypropylene centrifuge tube. Extraction with acetonitrile was repeated twice, and the combined extracts were defatted with 5 mL of n-hexane. Following centrifugation, the upper phase was discarded. The defatting step was then repeated twice, and the residue discarded. The SPE silica cartridge, used as a filter, was conditioned with 5 mL of acetonitrile and loaded with the sample extracts. The eluates were collected quantitatively in a 15 mL polypropylene centrifuge tube. Subsequently, for both matrices, 2 mL of acetonitrile was passed through the cartridge and the eluates were combined with the first one. Concerning muscle matrix only, a further 2 mL of acetonitrile was passed through the cartridge and combined and then partially evaporated to dryness (N_2_, 50 °C). The SPE were washed with 6 mL ethanol–water–ammonia (90 + 10 + 0.5 *v*/*v*/*v*) to quantitatively collect the analytes at the end of the filtration process, and then the combined solutions were brought to complete dryness with N_2_. Finally, the dry residues were redissolved in 1 mL of methanol, filtered and transferred into LC vials.

#### 2.3.2. Multi-Residue LC-MS/MS Method

The samples were analysed using a multi-residue LC-MS/MS method previously described by Galarini et al. [22] and Moretti et al. [23], enabling the determination of 11 coccidiostats in meat, eggs and feed samples. Chromatographic separation was performed on a Thermo Electron instrument (San Jose, CA, USA) consisting of a surveyor HPLC and a TSQ Quantum Ultra triple quadrupole mass spectrometer operating in both positive and negative ESI modes. The capillary temperature was 320 °C and the capillary voltages were set at 4.0 and 3.0 kV for positive and negative ionization, respectively. The Q1 and Q3 resolutions were set at 0.7 mass units FWHM. Sheath gas, auxiliary gas and ion sweep gas (N_2_) pressures were set at 30, 15 and 5 arbitrary units, respectively. Collision gas (Ar) pressure was 1.5 mtorr. The instrument was controlled by Xcalibur software (version 3.0, Thermo Fisher Scientific, Waltham, MA, USA). Analytes were separated on a Synergi Fusion HPLC column (150 × 2.0 mm, 4 µm; Phenomenex, Torrance, USA). HPLC mobile phase A was an aqueous solution containing 0.1% (*v*/*v*) formic acid, and mobile phase B was acetonitrile containing 0.1% (*v*/*v*) formic acid and 0.02 mM sodium acetate. The gradient was initiated at 15% eluent B and kept for 2 min, continued with a linear increase to 25% B in 1 min, followed by a linear increase to 95% B in 12.5 min. This percentage was maintained for 17.5 min. The system returned to 15% B in 2 min and the column was re-equilibrated for 5 min. The column temperature was 40 °C and the autosampler tray temperature was kept at 16 °C. The flow rate was 0.25 mL/min and the injection volume was 5 µL.

### 2.4. Method Validation

The method was validated according to Commission Decision 2002/657/EC [28] in food matrices, while for feed validation the scheme reported by Moretti et al. [23] was used. Both methods were included in the scope of ISO 17,025 accreditation [29]; the method performance has been successfully evaluated through participation in interlaboratory exercises organized by different international organizations such as the Food Analysis Performance Assessment Scheme (FAPAS), the Progetto Trieste-Proficiency Testing Schemes and the International European Union Reference Laboratory (EURL). The Z-scores in all cases were satisfactory (|z| ≤ 2.0).

The standard additions approach was used to quantify target analytes, therefore known amounts of the eleven coccidiostats were added to blank feed and food samples to obtain the target concentrations at seven levels (0.003, 0.005, 0.050, 0.10, 0.50, 1.0, 10 mg/kg) in feed and at five levels (1, 3.2, 10, 32, 100, 320 µg/kg) in food.

Selectivity/specificity, linearity, matrix effect, limit of determination (LOD), limit of quantification (LOQ), trueness (recovery), precision (repeatability and intra-laboratory reproducibility), decision limit (CCα), detection capability (CCβ), ruggedness (minor changes) and measurement uncertainty were evaluated. The experimental validation parameters are reported in Table 1.

### 2.5. Human Dietary Exposure and Risk Assessment

Dietary exposure (DE) was calculated as reported in literature [30,31]. Briefly, the results of coccidiostatic determination in food matrices (poultry meat and eggs) were combined with the food consumption data available from national Italian dietary surveys [32] at a detailed level, for the two mentioned food categories. Five age-based population groups were considered for the exposure assessment: infants/toddlers (0–2 years old), children (3–9 years old), adolescents (10–17 years old), adults (18–64 years old) and the elderly (65–97 years old). The body weight data for the groups considered, referring to the age classes set in the national survey, were 11, 26, 53, 70 and 70 kg, respectively [33]. A crucial step in DE evaluation, especially in chemical risk assessments, is managing data below the LOD. These data are referred to as non-detects, and the occurrence distribution is left-censored. During the present study, left-censored data was handled, as suggested in the literature for studies in the field of food safety, with the substitution method [34,35]: non-detect samples were replaced by zero in the lower bound (LB) approach and by LOD values in upper bound (UB), representing the worst and the best case scenarios. The risk assessment for Italian consumers was conducted by comparing the DE, estimated for high consumers (95th percentile of food consumption) with the ADIs. Dietary coccidiostat exposure through meat and hen eggs also was reported as a percentage contribution to ADI.

## 3. Results and Discussion

### 3.1. Results of Official Control: Targeted Samples

Concerning feedstuff, as reported in Table 2, during 2012–2017, 262 unmedicated feed samples were subjected to official control in the Umbria and Marche regions, the majority of which (72.1%) referred to poultry production, followed by bovine (11.4%), ovine (5.7%), rabbit (5.7%), swine (3.8%) and equine production (1.4%). The percentage of positive (above LOQ) feed samples ranged from 17.2% in 2012 to 28.3% in 2017, resulting in an average percentage of positive samples for the whole period tested of 25.6% (Table 2). Furthermore, 4.8% and 0.75% of total samples were found to be positive for two and three molecules, respectively.

Moretti et al. [23] found higher rates of multiple residue contamination in feed during 2010–2012, reporting 18%, 15%, 6% and 2% of tested samples simultaneously contaminated by two, three, four and five coccidiostats, respectively.

Concerning non-compliant samples, as shown in Table 2, the average percentage values moved from the 6.9% registered in 2012 to 1.7% in 2016; the following year (2017) the value recorded was 6.3%. It is to be noticed that the non-compliance rate varies mainly as a result of an increase in samples tested during the years examined, rather than an effective decrease in positive samples. Ionophoric molecules were detected in 28 feed samples (10.4%), while non-ionophoric molecules were found in 54 samples (20.15%); these results differ from those reported by Annunziata et al. [1] for ionophoric compounds (32.4%) and are comparable to those reported by the same authors in 2018 [36] for non-ionophoric residues in feed samples (20.3%) collected as part of the official Italian monitoring plan in other regions of Italy. Among ionophoric molecules, the most frequently detected coccidiostat in untreated feed was LAS, found in 11 samples, one of which exceeded the regulatory limit, followed by NAR found in 7 samples of which one was a noncompliant sample (Table 3). These results diverge from those reported by Annunziata et al. [1] and Moretti et al. [23] who found MON as the most frequently detected carry-over ionophore coccidiostat in feedstuff collected according to Italian NRCP (22% and 35%, respectively).

Among non-ionophoric anticoccidials, the most frequently detected compound was DIC, found in 27 samples, 3 of which were above the maximum residue level, followed by ROB that exceeded the regulatory limit in 1 out of 17 positive samples (Table 3). These results are in partial agreement with Moretti et al. [23] and with Annunziata et al. [36], whose studies also refer to ROB being the most frequently detected non-ionophoric molecule in feed samples.

Considering the results from different countries (Table 4), Pietruk et al. [37] analysed commercial feed in Poland during 2014 and found that coccidiostats were detected in 72.7% of samples analysed and 18% were contaminated with more than one analyte, showing higher rates of both positive and multi-molecule positive samples than those reported in this study. The authors also found that SAL was the most common ionophoric coccidiostat (45.5%), followed by NAR (18.2%) while, similar to the results presented in this study, DIC and ROB were the most frequently detected non-ionophoric molecules (12.1% and 9.1%, respectively). Moreover, a Belgian study by Delahaut et al. [14] reported levels of carry-over contamination of 15% of sampled feedstuff and detected mainly SAL, MON, NIC, ROB and NAR. Moreover, Agunos et al. [38] published a study about antimicrobial use surveillance in broiler chicken flocks in Canada during 2013–2015, in which SAL and NIC were the most frequently found ionophoric and chemical (non-ionophoric) coccidiostats in broiler feed, respectively, followed by the NAR–NIC combination. The authors also referred to seasonal variations observed in the use of coccidiostats. During summer, the most frequently reported coccidiostats were SAL, MAD and DEC, while in winter the most frequently reported coccidiostats were MON and NIC. As mentioned above, the occurrence of coccidiostats in feed due to cross-contamination may result in the presence of residues of these substances in food of animal origin, representing a potential threat to human health due to allergic reactions or to toxic effects. Particularly, the presence of coccidiostat residues in edible tissues and eggs has been reported in several countries since the late 1990s [39,40]. This has encouraged research and application of surveillance programs in many countries to monitor and prevent unacceptable contamination of food of animal origin [24]. Two hundred and two samples of meat of different species (5 bovine, 29 swine, 166 poultry, 1 ovine and 1 rabbit), and 151 of hen eggs were analysed for coccidiostat residues during 2012–2017 in the present study, in compliance with the NRCP. As for feed samples, poultry was the most represented species in the sampling plan, representing 82.2% of all meat samples and 97% of positive samples. During the time frame considered, positive samples of animal muscle ranged from 14.7% to 48.7%; the overall mean value of positive samples for the years considered was 33.7% (Table 2). Ionophoric molecules were detected in 6 meat samples (2.8%), and the most represented molecules were LAS and NAR; non-ionophoric compounds were found in 66 samples (31.7%) and, among these, the most frequently detected were NIC and DIC (Table 3). Contrary to what has been previously reported for feed, no meat samples were found positive for more than one coccidiostatic molecule, and the concentrations detected in no case exceeded the MRLs. The results presented differ from those reported by Moretti et al. [23] concerning the survey of food samples collected within the Italian Control Programme 2010–2012, according to which only 5% of meat samples were positive for coccidiostats, and NIC (9.6–44 μg/kg), ROB (2.1 μg/kg) and LAS (2.0 μg/kg) were the most frequently detected molecules.

Furthermore, a recent study in Greece regarding the presence of coccidiostats in animal tissue, revealed a positive rate (20.7%) close to the value reported here (Table 2), although a higher non-compliant rate (1.21%) was registered [24]. Furthermore, the authors reported that 47% of positive samples were found to be contaminated with more than one molecule, DEC, SAL and MAD being the most prevalent ones in animal tissues, mainly poultry muscle, suggesting that combined treatment may represent a common practice for Greek farmers while, as previously reported, no meat samples of the present study were found to contain more than one coccidiostat residue. Moreover, according to the latest EFSA report on the results from the monitoring of veterinary medicinal product residues and other substances in live animals and animal products [41], samples of non-compliant for anticoccidials were reported in pigs (0.04%, mainly for toltrazuril sulphone), poultry (0.12%, mainly for SAL) and rabbit (0.59%, mainly for SAL), describing a non-compliance rate that was not detected in the samples analysed in the present study. Theoretically, the use of coccidiostats for laying hens is, from a clinical point of view, not needed, because at that age animals are usually immunized. Nevertheless, laying hens are the most likely of the non-target species to be exposed to coccidiostats in practical conditions. This is due to the fact that traces of feeds for rearing chickens, for which coccidiostats are licensed until the age of 14 or 15 weeks, may remain in feed silos and, subsequently, be consumed by laying animals [25]. Data from this study reveal that the percentage of positive egg samples varied noticeably during the years considered, as reported in Table 2, from the lowest value recorded in 2014 (4.3%) to the highest recorded in 2015 (33.3%) and with an overall mean value of positive samples of 15.9%. Non-ionophoric compounds were detected in a larger number of samples in comparison to ionophoric molecules, 13.6% and 7.2% respectively; furthermore, NIC and ROB were the two most frequently found non-ionophoric molecules while LAS and MAD were the most detected coccidiostats of synthetic origin (Table 3). The presence of LAS in eggs has been reported since the 1990s; an Irish study (Table 4) estimated that 66% of eggs were positive in 1994, and despite substitution of the powdered formulation with a granular premix leading to a decrease in the incidence of LAS in eggs (21% in 1995), this molecule continues to be detected in both eggs and poultry, albeit in a lower proportion of cases [42]. During 2005, an EU survey (Table 4) indeed reported LAS to be the most frequent coccidiostat found in eggs in the UK, being detected in 22.6% of egg samples [43]. A factor that may contribute to the accumulation of LAS in eggs is the great affinity for egg yolk shown by the molecule [25,44]. As well as not being previously reported for meat, simultaneous positivity for more than one anticoccidial has not been recorded for egg samples either. Nevertheless, violative residues were detected in 2.2% of total samples analysed (3 samples, 1 in 2014 for DIC and 2 in 2015 for LAS) (Table 3). These results differ slightly from those reported by the EU-wide Surveillance on Coccidiostat Residues in Food report, in which hen eggs were attributed non-compliance rates of 1.2%, 0.72% and 0.54% for the years 2010, 2011 and 2015, respectively, as shown in Table 4 [45,46]. A slightly lower prevalence of contaminated egg samples (13%) was reported by Moretti et al. [23], referring to the above-mentioned Italian Control Programme, in which NIC (1–32 μg/kg), ROB (1.1–11 μg/kg), LAS (1.0–21 μg/kg) and DIC (2.1 μg/kg) were the most frequently recovered anticoccidial compounds in eggs, albeit always under the acceptable limits. Furthermore, Dubreil–Chéneau et al. [47] published the results of a French monitoring plan conducted in 2007 that differ noticeably to those reported in this study. The authors reported that 49% of egg samples analysed were positive for one or more anticoccidials (Table 4), mainly NIC (18%), DIC (7%), ROB (7%) and MAD (1%); 29% of positive samples were found to be non-compliant. Seen in the above-mentioned EFSA report [41], a lower percentage (0.81%) of egg samples was found to exceed anticoccidial reference limits, and the majority of violative samples were found to be positive for SAL and DIC, among ionophores and non-ionophores, respectively. As shown, data concerning the presence of coccidiostats in feed and food attributable to unavoidable carry-over presents a certain degree of variability; furthermore, comparison of the results with those in the literature may be difficult due to the lack of detailed information on the range of coccidiostats subjected to control, types of matrices analysed and established limits, especially for non-EU members. Even within the EU, the scope for residue control varies among different member states, as do breeding practices. Moreover, it should be noted that the majority of the legislation concerning maximum limits for coccidiostats in food of animal origin has been revised in the last few years; therefore, according to the law in force at a specific time, the non-compliance rate may vary [40].

### 3.2. Human Dietary Exposure and Risk Assessment

Concerning coccidiostats, an appropriate measure for the potency of the substance is the ADI: the amount of a substance in food that can be ingested on a daily basis over a lifetime without an appreciable health risk. Therefore, ADI values provide a measure of safety during long-term exposure to repeated ingestion of substances in foods [48]. To estimate the DE to coccidiostats, food consumption data were collected.

Data show that daily consumption of meat decreases with increasing age and ranges from 4.35 g/kg bw/kg for toddlers to 1.07 g/kg bw/kg for the elderly aged 65–97 years. Similarly, daily egg consumption decreased with increasing age, from 1.99 g/kg bw/kg for the 0–2 years population to 0.87 g/kg bw/kg for the elderly, with the exception of the group of 3- to 9-years-old children (2.64 g/kg bw/kg) representing the highest value. Combining data from food consumption surveys with the analytical data of coccidiostat residues in food, the risk for human health related to the consumption of positive foodstuffs was assessed, and the contribution to ADI was calculated. Data reveal that the exposure to coccidiostats through the consumption of meat represents less than 1% of the ADIs for all the molecules and for all the age categories considered, for both LB and UB. As mentioned above, laying hens are the most likely of the non-target species to be exposed to coccidiostats; therefore, residual levels of such molecules in eggs have to be considered, in addition to possible tissue levels in target and non-target animal species, in a human exposure assessment [25]. Similarly, the intake of coccidiostats through hen eggs represents less than 1% of the ADIs for all the age categories considered, and for both LB and UB scenarios. Results indicate that the direct public health risk represented by the intake of coccidiostats via meat and egg consumption is negligible. Similar conclusions have been reported by Dorne et al. [25] who reported that no appreciable risk for consumers could be identified for LAS, MAD, MON, NAR, NIC, SAL, DEC, DIC and ROB following the consumption of meat, offal or eggs derived from non-target animals. Another study developed by Bacila et al. [7] reports that the consumption of usual daily quantities of poultry products, considering the ADIs, is unlikely to pose any risk to human health related to NIC, one of the most used anticoccidials in poultry production. These considerations on health risk posed by coccidiostatic residues also were corroborated by Kadykalo et al. [5] who analyzed several aspects of the problem. The authors concluded that, even though the residue level of zero is not achievable, the withdrawal times applied by the poultry industry will result in residues below the MRL, effectively eliminating any potential human risk in poultry; furthermore, even when cross-contamination of anticoccidials occurs, it does not represent a threat to human health because the exposure remains below the value needed to exceed the ADI.

Other studies have considered the risk to human health due to coccidiostat residues present in contaminated vegetables [49]. The authors provided information on the DE of consumers to coccidiostats via vegetables grown with contaminated poultry manure, demonstrating that some plants, such as carrots and lettuce, are capable of taking up coccidiostats from the soil. Nevertheless, the authors also reported that coccidiostats in vegetables, due to the low incorporation levels coupled with food consumption data, are unlikely to represent a threat to public health, as proved by the low contribution to ADI (around 1%).

## 4. Conclusions

Reported data indicate that in the central Italy context, contamination of untreated feedstuffs as a consequence of carry-over occurs widely for almost all animal productions, leading indirectly to the presence of coccidiostat residues in foodstuffs. Evidence shows that poultry production (egg and meat) presents the higher rate of positive samples, although the related public health risk represented by the intake of anticoccidials is negligible, as confirmed by the low contribution to ADIs. The results obtained also suggest that the monitoring for residues in food and feed, the establishment of safe residue limits, and risk assessment studies are well-handled by the authorities, therefore resulting in effective strategies for ensuring the safety of food production where anticoccidials are used.

## Figures and Tables

**Table 1:** Validation parameters for the methods used in the determination of coccidiostats in feed, muscle and eggs for residue control in Italy.

Analyte	Matrix	LOD (µg/kg)	LOQ (µg/kg)	CV Repeatability (%)	CV Reproducibility (%)	Apparent Recovery Range (%)	Relative Expanded Uncertainty (%)	CCα (µg/kg)	CCβ (µg/kg)
NIC	Feed	3	5	4.7	5.7	87–119	20.7		
	Eggs	1	1	3	4.7	83–115		323	348
	Poultry	1	1	2.1	5.4	86–115		4354	4740
	Other species	1	1	2.1	5.4	86–115		54	59
ROB	Feed	3	5	3.6	5.4	87–119	20.4		
	Eggs	1	1	4.8	6.9	83–115		28	31
	Poultry, rabbit	1	1	5.5	9.1	86–115		230 ^a^	264 ^a^
	Other species	1	1	5.5	9.1	86–115		5.7	6.6
DIC	Feed	3	5	5.1	6.3	87–119	21.4		
	Eggs	1	1	6.3	7.8	83–115		2.3	2.5
	Poultry	1	1	4.2	8.5	86–115		570	649
	Rabbit	1	1	4.2	8.5	86–115		171	195
	Other species	1	1	4.2	8.5	86–115		5.7	6.5
HFG	Feed	3	5	6.5	9.7	87–119	25.9		
	Eggs	1	1	14	20.6	83–115		8	11
	Bovine	1	1	9.5	14.4	86–115		12	15
	Other species	1	1	9.5	14.4	86–115		3.7	4.6
DEC	Feed	3	5	3.4	5.3	87–119	20.3		
	Eggs	1	1	2.2	5.0	83–115		22	23
	Poultry	1	1	2.3	10.7	86–115		588	691
	Other species	1	1	2.3	10.7	86–115		24	28
LAS	Feed	3	5	5.2	6.3	87–119	21.4		
	Eggs	1	1	4.5	9.6	83–115		174	201
	Poultry	1	1	4.5	9.4	86–115		69	80
	Bovine	1	1	4.5	9.4	86–115		12	13
	Other species	1	1	4.5	9.4	86–115		5.7 ^b^	6.6 ^b^
MON	Feed	3	5	4.4	6.9	87–119	22.1		
	Eggs	1	1	4.3	6.2	83–115		2.2 ^c^	2.4 ^c^
	Bovine	1	1	4	6	86–115		2.2	2.4
	Poultry	1	1	4	6	86–115		8.8 ^c^	9.6 ^c^
	Other species	1	1	4	6	86–115		2.2 ^c^	2.4 ^c^
SAL	Feed	3	5	4.5	6.2	87–119	21.3		
	Eggs	1	1	5.3	7	83–115		3.3 ^d^	3.7 ^d^
	Poultry	1	1	3.5	6.1	86–115		16.5 ^d^	18.1 ^d^
	Other species	1	1	3.5	6.1	86–115		2.2 ^d^	2.4 ^d^
NAR	Feed	3	5	4.9	7.2	87–119	22.6		
	Eggs	1	1	5.6	7.3	83–115		2.2	2.5
	Poultry	1	1	3.7	60	86–115		55	60
	Other species	1	1	3.7	60	86–115		5.5	6
SEM	Feed	3	5	7	9.6	87–119	25.8		
	Eggs	1	1	8.7	15.1	83–115		2.5	3.1
	Other species	1	1	6.6	10.5	86–115		2.3	2.7
MAD	Feed	3	5	6.6	9.2	87–119	25.3		
	Eggs	1	1	11.6	17.2	83–115		15	20
	Poultry	1	1	6.7	11.4	86–115		36 ^e^	43 ^e^
	Other species	1	1	6.7	11.4	86–115		2.4	2.8

^a^ ROB hydrochloride, ^b^ LAS sodium, ^c^ MON sodium, ^d^ SAL sodium, ^e^ MAD ammonium.

**Table 2:** Results for residue control of coccidiostats in central Italy during 2012–2017 in feed and food (muscle and eggs).

	2012			2013			2014			2015			2016			2017			Total		
	No. Tested	No. Positive	No. Nc	No. Tested	No. Positive	No. Nc	No. Tested	No. Positive	No. Nc	No. Tested	No. Positive	No. Nc	No. Tested	No. Positive	No. Nc	No. Tested	No. Positive	No. Nc	No. Tested	No. Positive	No. Nc
Feed																					
Bovine	3	1		3			3			6			10	2		5	1		30	4	
Swine	1			2			1			1			1			4	2	1	10	2	1
Equine										1			1			1	1	1	3	1	1
Poultry	20	2	1	21	5	2	22	7	1	50	13	1	44	12	1	32	8	1	189	47	7
Ovine	2			3			3			3	1		2			2			15	1	
Rabbit	3	2	1	1	1		1	1		6	6		2	1		2	1		15	12	2
**Total**	29	5	2	30	6	2	30	8	1	67	20	1	60	15	1	46	13	3	262	67	10
%		17.2	6.9		20.0	6.7		26.7	3.3		29.9	1.5		25.0	1.7		28.3	6.3		25	3.8
Food																					
Bovine				2			1			1			1						5		
Swine	4			8			4			4			4			5			29		
Poultry	28	3		31	12		33	14		6	1		34	19		34	19		166	68	
Ovine	1	1																	1	1	
Rabbit	1	1																	1	1	
**Total**	34	5		41	12		38	14		11	1		39	19		39	19		202	70	
%		14.7			29.3			36.8			9.1	-		48.7			48.7			33.7	
Eggs	20	3		18	3		47	2	1	30	10	2	24	4		12	2		151	24	3
%		15.0			16.7			4.3	2.1		33.3	6.7		16.7			16.7			15.9	2.0

The total number of tested samples is presented.

**Table 3:** Incidence of positive and noncompliant (n.c.) samples, residue concentration range in feed and food matrix collected within the Italian National Residue Control Plan during 2012–2017.

		Feed						Food (Hen Eggs and Meat)			
		Positive Samples (n.c)	Incidence (%) *	Min–Max (mg/kg)	Specie Involved			Positive Samples (n.c)	Incidence (%)	Min–Max (µg/kg)	Specie Involved
2012						2012					
	LAS	2	6.9	0.2	Poultry, Rabbit		NIC	4	7.4	1.4–9.6	Poultry, Ovine, Eggs
	DIC	3 (1)	10.3 (3.4)	0.011–0.081	Rabbit		LAS	2	3.7	1.5–2.0	Poultry, Eggs
	NAR	1	3.4	0.1	Bovine		ROB	1	1.9	1.3	Rabbit
	ROB	1 (1)	3.4	0.9	Poultry		MAD	1	1.9	1.5	Eggs
2013						2013					
	LAS	1	3.3	0.5	Poultry		NIC	9	15.3	1.2–21	Poultry, Eggs
	ROB	5 (1)	16.7	0.2–2.0	Poultry, Rabbit		LAS	2	3.4	2.5–8.2	Poultry
	MON	1 (1)	3.3 (3.3)	4	Poultry		ROB	1	1.7	1.8	Eggs
	SAL	1	3.3	0.1	Poultry		DIC	2	3.4	3.3–7.4	Poultry
							SAL	1	1.7	3	Eggs
2014						2014					
	LAS	1	3.3	0.4	Poultry		NIC	14	16.5	1.3–238	Poultry, Eggs
	DIC	4 (2)	13.3 (6.7)	0.007–0.038	Poultry, Rabbit		LAS	1	1.2	1.2	Eggs
	ROB	4	13.3	0.1–0.6	Poultry		ROB	1	1.2	1.6	Poultry
	MAD	1	3.3	0.003	Poultry		DIC	1 (1)	1.2 (1.2)	2.4	Eggs
2015						2015					
	LAS	2 (1)	3.0 (1.5)	0.4–5.9	Poultry		LAS	4 (2)	9.8 (4.9)	2.8–1002	Eggs
	DIC	10	14.9	0.003	Poultry, Rabbit, Ovine		ROB	7	17.1	1.2–4.7	Poultry, Eggs
	NAR	3	4.5	0.6–2.1	Poultry		DIC	1	2.4	1.2	Eggs
	ROB	4	6.0	0.3–0.8	Poultry, Rabbit		DEC	1	2.4	1.8	Eggs
	SAL	1	1.5	0.4	Rabbit						
	MAD	1	1.5	0.05	Poultry						
	DEC	3	4.5	0.2–0.7	Ovine, Rabbit						
	NIC	2	3.0	0.2–0.8	Poultry						
2016						2016					
	LAS	4	6.7	0.1–1.1	Poultry		NIC	20	31.7	1.3–516	Poultry, Eggs
	DIC	6	10.0	0.007–0.038	Poultry, Bovine		LAS	1	1.6	26	Eggs
	ROB	2	3.3	0.2	Poultry, Rabbit		NAR	1	1.6	1.3	Poultry
	MON	2 (1)	3.3 (1.7)	0.3–1.8	Poultry		DEC	1	1.6	70	Poultry
	MAD	1	1.7	0.003	Poultry						
	DEC	1	1.7	0.4	Bovine						
2017						2017					
	LAS	1	2.2	0.4	Swine		NIC	13	25.5	1.0–32.1	Poultry, Eggs
	DIC	4	8.7	0.004–0.014	Poultry, Rabbit		DIC	4	7.8	1.4–11	Poultry
	NAR	3 (1)	6.5 (2.2)	0.1–56	Poultry		DEC	2	3.9	2.5–5.4	Eggs
	ROB	1	2.2	0.2	Poultry		MAD	1	2.0	1	Eggs
	MON	1	2.2	0.1	Poultry		NAR	1	2.0	3.4	Poultry
	MAD	1	2.2	0.007	Poultry						
	DEC	3 (2)	6.5 (4.3)	0.3–4.6	Equine, Swine, Bovine						
	NIC	2	4.3	0.1–0.4	Poultry						

* Incidence was calculated referring to the total samples tested in each year as reported in Table 2.

**Table 4:** Positive samples recorded from different countries.

Country	Year	Matrix	Coccidiostat	Most Frequently Detected Coccidiostat	% Positive	Reference
Poland	2014	Feed	various	SAL	72.7	Pietruk et al. [37]
Belgium	2009	feed	various	SAL	15	Delahaut et al. [14]
Canada	2013–2015	Feed	various	SAL	n.d.	Agunos et al. [38]
Italy	2010–2012	feed	various	MON	62	Moretti et al. [23]
Greece	2016	Food	various	DEC, SAL, MAD	20.7	Dasenaki et al. [24]
Ireland	1996	Food	LAS	LAS	66	Kennedy et al. [42]
UK	2005	Food	various	LAS	35.6	Mortier et al. [43]
France	2007	Food	various	NIC	49	Dubreil–Chéneau et al. [47]
Italy	2010–2012	Food	various	NIC	9	Moretti et al. [23]
Europe	2010	Food	various	n.d.	1.2	EU Commission [45]
	2011	Food	various	n.d.	0.72	EU Commission [45]
	2015	Food	various	n.d.	0.54	EU Commission [46]

n.d. not defined.
